# Prevalence and antifungal susceptibility of Candida albicans causing vaginal discharge among pregnant women in Lebanon

**DOI:** 10.1186/s12879-019-4736-2

**Published:** 2020-01-13

**Authors:** Nahed Ghaddar, Elie Anastasiadis, Rawad Halimeh, Ali Ghaddar, Rita Dhar, Wadha AlFouzan, Hoda Yusef, Mira El Chaar

**Affiliations:** 1 Faculty of Science, Biological Sciences Department, Beirut Arab University, Beirut, Lebanon; 2 Department of Obstetrics and Gynecology, Saint George Hospital, Beirut, Lebanon; 3 Faculty of Medicine, University of Balamand, Beirut, Lebanon; 4 Department of Biomedical Sciences, Lebanese International University, Beirut, Lebanon; 5 Microbiology Unit, Department of Laboratories, Farwania Hospital, Sabah Al Nasser, Kuwait; 6 Department of Microbiology, Health Sciences Center, Kuwait University, Jabriya, Kuwait; 7 Faculty of Health Sciences, University of Balamand, P.O.Box 166378 Ashrafieh, Beirut, 1100-2807 Lebanon

**Keywords:** *Candida albicans*, Vulvovaginitis, Prevalence, Pregnant women, Antifungal susceptibility

## Abstract

**Background:**

Vaginal candidiasis is frequent in pregnant women and is associated with sepsis and adverse neonatal outcomes. This study determined the prevalence of *candida* species in symptomatic pregnant women and evaluated the antifungal susceptibility profile of the isolated *Candida* strains. It also aimed to explore whether *Candida* species predicts gestational complications and adverse neonatal outcomes.

**Methods:**

A total of 258 pregnant women with vaginal discharge at 35 to 37 week of gestation participated in this study. Vaginal swabs from these patients were collected at various obstetrics and gynecology clinics in Lebanon for a period of 14 months. *Candida* isolates were identified at species level and antifungal susceptibility of *Candida albicans* to fluconazole (FCZ), amphotericin B (AMB), itraconazole (ICZ) and voriconazole (VCZ) was determined by the agar-based E-test method.

**Results:**

Among 258 women tested, 100 (39%) were positive for *Candida* species. *C. albicans, C. glabrata* and *C. krusei* were isolated from 42, 41 and 17% of the women, respectively. *C. albicans* was significantly associated only with gestational diabetes while *C. krusei* or *C. glabrata* had significant positive associations with other gestational complications. The antifungal susceptibility tests of *C. albicans* isolates revealed 97.5, 90, 87.5 and 97.5% susceptibility to AMB, FCZ, ICZ and VCZ, respectively.

**Conclusion:**

The current study revealed high incidence of both *C. albicans* and non-*C. albicans Candida* strains causing vulvovaginitis among pregnant women in Beirut, Lebanon. *Candida* screening as antenatal follow up is advised to minimize the risk of adverse neonatal outcome or gestational complications.

## Background

*Candida* species, which are part of the normal flora in the vulvovagina, may cause opportunistic infections under various circumstances that compromise host immunity. *Candida* spp. subsist in symbiotic relationship with vaginal microbiota, therefore asymptomatic colonization is common and may persist for years. The rate of genital *Candida* colonization ranges from 20% in asymptomatic young women to up to 30% in pregnant women [1–6]. The risk factors associated with increased rate of vulvovaginal candidiasis (VVC) in pregnant women are immunologic alterations, increased estrogen levels, and increased vaginal glycogen production mechanism [3].

VVC is the result of *Candida albicans* in 85–95% of cases whereas incidence rate of Non-*C. albicans Candida* (NCAC) in pregnant women and non-pregnant women is less than 10% as described in previous studies [7].

Treatment of VVC is recommended only in the presence of symptoms since over 20% of women may have yeast as part of their natural vaginal microbiome and are asymptomatic [8]. However, pregnant women may have severe and prolonged symptoms of VVC requiring longer courses of therapy [9]. Recent studies have shown an increase in the development of drug-resistance among *C. albicans*, less is known about the burden and effects of drug resistant fungal infections.

Candidiasis in newborns has been associated with increased risk of pregnancy complications, such as premature rupture of membranes, preterm labor, chorioamnionitis, and congenital cutaneous candidiasis. Colonization with *Candida* spp. in neonates may occur by vertical transmission from the mother during the perinatal period or by horizontal transmission in the nursery or the neonatal intensive care unit (NICU) [10–12]. It has been shown that 5 to 30% of all colonized preterm neonates will develop invasive *Candida* infection (ICI) during their stay in the NICU [13–15]. *C. albicans* was shown to play a major role in neonatal colonization in the first days of life and were also documented in a group of premature infants [16].

The epidemiology of antifungal resistance among *C. albicans* in pregnant women in Lebanon remains poorly reported. Therefore, the objective of this study was to determine the prevalence of *Candida* species in symptomatic pregnant women with vaginal discharge at 35 to 37 weeks of gestation and to evaluate the antifungal susceptibility profile of the isolated strains of *C. albicans*. In addition, the study evaluated the association between the presence of *Candida* species and gestational complications and outcomes.

## Methods

### Study sample and procedure of collection

In this study, a cross-sectional design was adopted for determining the prevalence of *Candida* species in Lebanese pregnant women. Clinical samples were collected from 258 pregnant women with vaginal discharge in three obstetrics and gynecology clinics in Lebanon during a period of 14 months (June 2015–July 2016). Women were approached by a registered gynecologist who explained the objectives of the study and asked them to participate. Participation was voluntary and anonymous. Two vaginal swabs were collected from each patient. The samples were stored in Stuart media (Oxoid, UK) at room temperature and transported to the clinical diagnostic laboratory.

### Data collection

Socio-demographic data, clinical status and gestational history of 165 (64%) patients were collected through a questionnaire that included information about mothers’ risk factors for adverse neonatal outcomes such as gestational diabetes, previous miscarriage, anemia and recurrent urinary tract infections (UTI). The 165 participants were followed up after delivery to gather information about delivery time, delivery type, induced labor, gestational complications (intrahepatic cholestasis, mitral valve prolapse, asthma, hypothyroidism, oligohydramnios and gestational thrombocytopenia) and neonatal outcomes (newborn height, weight and apgar score).

### Literature search

**Fig. 1:**
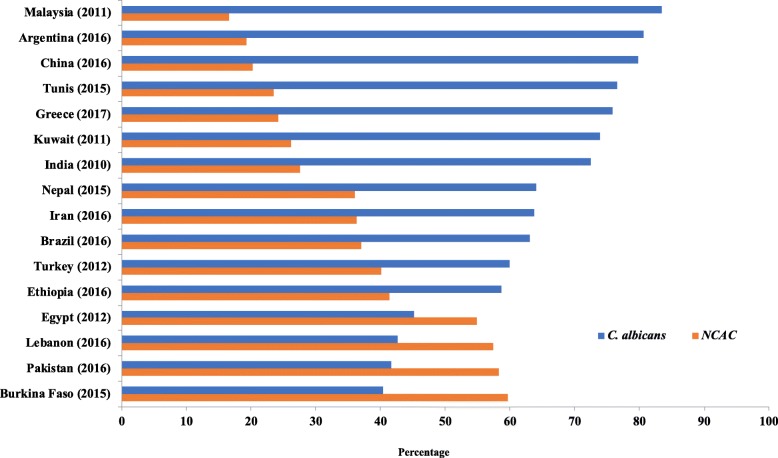
Distribution of *Candida* species isolated from vaginal swabs of women from various countries after 2010

To compare the distribution of *Candida* species isolated from women genital tract in different countries, a Pubmed search was performed that included articles published in the last 10 years [17–23]. Fifteen articles were selected and summarized in Fig. 1.

### Culture and identification

Vaginal swabs were cultured on both Sabouraud dextrose agar (SDA) and Chromatic *Candida* medium (Liofilchem, Italy). The latter allows the selective isolation and differentiation of *Candida* spp. based on colony color and morphology; it has been well documented in previous studies as for its high sensitivity and specificity for the identification of the most commonly encountered *Candida* spp. [24–26]. Both plates were incubated at 37 °C for 48 to 72 h. The chromatic characteristics of the colonies were the following: green colonies were identified as *C. albicans*, creamy colored colonies as *C. glabrata*, and pink with a whitish border colonies as *C. krusei*. All isolates were confirmed by API 20 C AUX strip (BioMerieux, Marcy l’Etoile, France). Further phenotypic testing was done to differentiate between *C. dubliniensis* and *C. albicans* by growing the germ tube positive yeast isolates at 45 °C on SDA for up to 10 days. *C. albicans* isolates were identified by their ability to grow at 45 °C.

### Antifungal susceptibility testing

The in vitro activity of the antifungal agents against each isolate was determined by the E-test (HiMedia, Mumbai, India) in accordance with the manufacturer’s instructions. The E-test strips of fluconazole (FCZ; 0.016 ∼ 256 μg/mL), itraconazole (ICZ; 0.002 ∼ 32 μg/mL), voriconazole (VCZ; 0.002 ∼ 32 μg/mL), and amphotericin B (AMB; 0.002 ∼ 32 μg/mL) were used [27]. Interpretive susceptibility criteria for antifungal breakpoints were adapted from the Clinical and Laboratory Standards Institute (CLSI), 2017 (21). The breakpoints used for *C. albicans* are: FCZ (S ≤ 2; SDD = 4; R ≥ 8); VCZ (S ≤ 0.12; R ≥ 1), ICZ (S ≤ 0.12; R ≥ 1) and AMB (S ≤ 2; *R* > 2). For quality control, *C. albicans* (ATCC 10231) was used as reference strain and tested simultaneously with the clinical isolates.

### Statistical analysis

The presence of *Candida* species was correlated with the newborn height, weight and Apgar score (overall assessment of new born well-being used immediately following the delivery of the baby) as dependent variables using linear multiple regression analysis. The models took into consideration to control other possible confounding effect of various independent variables including mother’s age, mother’s education, delivery type, delivery week, induced labor, recurrent urinary tract infection (UTI), gestational diabetes, anemia and other gestational complications. Statistical significance was calculated using *p*-value and confidence intervals. The presence of *Candida* species effect on categorical outcome variables (Gestational diabetes, vaginal discharge, induced labor and recurrent UTI) was explored by comparing frequencies using the test of independence Chi-square. *P* values were computed considering *p* ≤ 0.05 as significant results.

## Results

The socio-demographic characteristics of 165 respondents are summarized in Table 1; 49% of women who participated were between the ages of 36 to 40 years old. The majority completed their university degree (62.4%). The rate of normal vaginal delivery was 43.6 and 69.7% of women had labor induction. Gestational complications occurred in 59.1% of women and 25.5% of women had gestational diabetes mellitus. Anemia was reported in 14.5% of women.

**Table 1:** Socio-demographic characteristics of 165 respondents

Age group	20–25 years	19 (11.5%)
	26–30 years	14 (8.5%)
	30–36 years	49 (29.7%)
	36–40 years	81 (49.1%)
	≥41 years	2 (1.2%)
Education status	Primary	32 (19.4%)
	Secondary	30 (18.2%)
	University	103 (62.4%)
Delivery type	Normal	72 (43.6%)
	Cesarean section	24 (14.5%)
	Missing	69 (41.8%)
Induced labor	Yes	50 (30.3%)
	No	115 (69.7%)
Recurrent UTI	Yes	4 (2.4%)
	No	161 (97.6%)
Gestational complications	Yes	97 (59.1%)
	No	67 (40.9%)
	Missing	1 (0.6%)
Gestational diabetes mellitus	Yes	42 (25.5%)
	No	123 (74.5%)
Anemia	Yes	24 (14.5%)
	No	141 (85.5%)

The data presented in the table are number (%) of patients. Missing are missing data since patients’ information were not provided; those were excluded from the analysis. Gestational complications include intrahepatic cholestasis, mitral valve prolapse, asthma, hypothyroidism, oligohydramnios and gestational thrombocytopenia.

Among the cultures from the 258 women tested, 100 (39%) were positive for *Candida* species.

*C. albicans* was isolated from 42% of the women (*N* = 42) and NCAC from the remaining 58%. Figure 1 summarizes the distribution of Candida species isolated from vaginal swabs from population-based studies conducted in different countries including our study over the last decade. China, Brazil, Tunis, Kuwait, India, Greece and Turkey have reported that *C. albicans* remains the most commonly isolated yeast (60–80%) in women diagnosed with VVC [17–23]. On the other hand, an increasing trend in the occurrence of NCAC (58–60%) over time has also been observed in Pakistan and Burkina Faso [28, 29]. The main identified NCAC in our study were *C. glabrata* (71%, *N* = 41) followed by *C. krusei* (29%, *N* = 17). Four women were co-infected with both *C. albicans* and *C. glabrata*. All of the three identified *Candida* species were isolated from women in the age group 30–40 years.

The observed susceptibility rates of *C. albicans* isolates to AMB, FCZ, ICZ and VCZ were 97.5, 90, 87.5 and 97.5%, respectively. MIC_50_ and MIC_90_ of the antifungal agents tested against 40 strains of *C. albicans* are presented in Table 2. Two of the isolates were lost during processing, Although ICZ presented the lowest MIC_90_ value of 0.125 μg/mL, it showed highest resistance rate (12.5%) among all the agents tested.

**Table 2:** Ranges of MICs, MIC50 and MIC90 and percentage resistance in 40 *C. albicans* isolates

Antifungal drugs	MIC 50	MIC 90	MIC Range	Percentage resistance
Amphotericin B	0.5	1	0.38–3.00	2.5%
Fluconazole	2	6	0.047–32	10.0%
Itraconazole	0.125	0.125	0.032–32	12.5%
Voriconazole	0.094	1	0.032–256	2.5%

MIC_50_ = Minimum Inhibitory Concentration required to inhibit the growth of 50% of organisms. MIC_90_ = Minimum Inhibitory Concentration required to inhibit the growth of 90% of organisms. MIC range is the range of the lowest and highest MIC values obtained from 40 *C. albicans* isolates tested*.* Percentage resistance is the percentage of isolates resistant to a specific antifungal drug.

The association between the presence of *Candida species,* isolated from 48 women who filled the questionnaire, was assessed with preterm delivery, delivery type, gestational complications, gestational diabetes, recurrent UTI infection and induced labor (Table 3). *C. albicans* was significantly associated only with patients with gestational diabetes; 33% of *C. albicans* positive and 24% of NCAC participants had gestational diabetes (*p* = 0.04). *C. albicans* had non-significant associations with gestational complications, induced labor and recurrent UTI. On the other hand, the presence of *C. krusei and C. glabrata* had strong significant association with premature delivery and gestational complications (*p* < 0.05): 94% of women with *C. glabrata* and 71.4% of women with *C. krusei* had gestational complications compared to 28.4 and 29.7% of women who did not have *C. glabrata* and *C. krusei* respectively (*p*-value ≤0.001). No significant associations were observed between the isolated *Candida* species and induced labor or recurrent UTI (Table 3).

**Table 3:** Association between *candida* species isolated from vaginal swabs of pregnant women and various clinical outcomes (*n* = 165)

		Delivery week (preterm delivery)		Delivery Type (C-section)		Gestational Diabetes n (%)		Gestational Complications n (%)		Induced Labor n (%)		Recurrent urinary track infection n (%)	
*C. albicans*	Yes *n* = 24	15 (62.5%)	*p* = 0.57	5 (20.1%)	*p* = 0.76	8 (33.3%)	*p* = 0.04	10 (41.7%)	*p* = 0.93	8 (33.3%)	*p* = 0.72	1 (4.2%)	*p* = 0.55
	No *n* = 141	72 (51.1%)		19 (13.5%)		34 (24.1%)		57 (40.7%)		42 (29.8%)		3 (2.1%)	
*C. glabrata*	Yes *n* = 17	13 (66.5%)	*P* = 0.02	6 (35.3%)	*p* = 0.32	6 (35.3%)	*p* = 0.13	16 (94.1%)	*p* ≤ 0.001	6 (35.3%)	*p* = 0.54	1 (0.06%)	*p* = 0.52
	No *n* = 148	63 (42.6%)		38 (25.7%)		33 (22.3%)		42 (28.4%)		41 (27.7%)		2 (0.01%)	
*C. krusei*	Yes *n* = 7	5 (71.4%)	*P* = 0.01	4 (57.1%)	*p* = 0.24	2 (28.5%)	*p* = 0.19	5 (71.4%)	*p* ≤ 0.001	3 (42.8%)	*p* = 0.46	1 (14.3%)	*p* = 0.43
	No *n* = 158	55 (34.8%)		61 (38.6%)		33 (20.8%)		47 (29.7%)		48 (30.3%)		4 (0.02%)	

*p* value < 0.05 was considered significant

Results of the three multiple regression models with neonatal outcomes (weight, height and Apgar score) as dependent variables are displayed in Table 4. Results revealed significant positive association between delivery time and neonatal height and significant negative association between C-section and height. Height increased 0.41 cm with 1 week increase in delivery time (*p* = 0.001) and decreased 0.46 cm with C-section (*p* = 0.002). Height also decreased with the presence of all identified *Candida* species. This reduction was statistically significant in both *C. krusei or C. glabrata infections* (Beta = − 0.46, *p* = 0.05 for *C. albicans* and Beta = − 0.77; *p* = 0.006). The other covariates did not yield significant associations with height. Neonatal weight had significant positive association with delivery time and significant negative association with C-section. There was 0.32 g increase in weight with an additional delivery week (*p* = 0.01) and 0.34 g decrease in weight with C-section (*p* = 0.02). Although weight decreased with the presence of *Candida* species (*C. albicans*: Beta = 0.16, *C. krusei* or *C. glabrata*: Beta = 0.43), this reduction was not statistically significant. The other covariates did not yield significant associations with weight. Apgar score did not show significant correlation with the presence of any *Candida* species or with any of the other independent variables.

**Table 4:** Effect of different variables on the height, weight and Apgar score of the neonates

	Height			Weight			Apgar Score		
	Beta	*p*-value	C.I	Beta	*p*-value	C.I	Beta	*p*-value	C.I
Age (≥41 years)	− 0.15	0.15	−1.44; 0.22	0.05	0.64	− 177.75; 284.12	− 0.105	0.385	−0.432; 0.16
Previous Miscarriage	0.03	0.79	−0.79; 1.03	−0.01	0.92	− 264.92; 241.54	0.15	0.20	−0.11; 0.54
Domestic Animals	0.11	0.30	−0.62; 1.97	−0.07	0.51	− 477.07; 242.68	− 0.04	0.72	− 0.59; 0.41
Delivery Week	0.41	0.001	0.34; 1.34	0.32	0.01	36.97; 311.59	0.01	0.94	−0.17; 0.18
Delivery Type	−0.46	0.002	−3.30; − 0.73	− 0.34	0.02	− 755.78; −45.68	− 0.16	0.29	−0.69; 0.21
Induced Labor	−0.09	0.48	−1.38; 0.65	−0.07	0.61	− 353.76; 209.41	− 0.04	0.76	− 0.41; 0.30
Gestational Diabetes mellitus	0.11	0.27	−0.39; 1.39	0.003	0.98	− 243.86; 249.62	0.10	0.37	−0.17; 0.46
Other gestational complications	0.18	0.10	−0.14; 1.68	0.25	0.04	16.61; 523.28	0.05	0.67	−0.26; 0.40
Anemia	−0.01	0.94	−1.20; 1.13	0.029	0.80	− 281.52; 364.01	− 0.005	0.96	−0.42; 0.40
Recurrent UTI	0.19	0.07	−0.23; 4.38	0.23	0.03	33.84; 1310.25	−0.12	0.29	−1.26; 0.38
*Candida albicans* infection	−0.46	0.05	−4.84; 0.08	−0.16	0.53	− 896.85; 465.31	0.09	0.73	−0.72; 1.02
*Candida glabrata/ krusei* infection	−0.77	0.006	−6.14; −1.04	−0.43	0.14	− 1228.04; 181.31	0.19	0.52	−0.61; 1.20
	*R*^2^ = 0.32			*R*^2^ = 0.25			*R*^2^ = 0.17		

Standardized beta coefficients (Beta) for each individual independent variable was calculated to compare the strength of the effect of each to the dependent variable. The higher the absolute value of the beta coefficient, the stronger the effect. Coefficient of determination (R2) was calculated to evaluate the proportion of the variance in the dependent variable that is predictable from the independent variables. The coefficient of determination assesses how well the model explains and predicts future outcomes. Confidence interval (CI) is the margin of error of the Beta. *P* value determines the significance of the results. *P* value < 0.05 was considered significant

## Discussion

The prevalence of *Candida* species causing vaginitis is pregnant women vary from one population to another. In our study, 39% of participating women were infected by *Candida* species. NCAC were more frequently isolated (58%) than *C. albicans* (42%). NCAC were also shown to increase in non vaginal clinical samples isolated from Lebanon; that was observed in a previous retrospective study published where the authors have shown that among all *Candida* strains isolated, *C. albicans* rates had decreased from 86% in 2005 to around 60% in 2014. However, the NCAC rates increased from 14% in 2005 to around 40% in 2014, comprising mainly of *C. tropicalis*, *C. glabrata*, and *C. parapsilosis* [30]. Recent emergence of NCAC, such as *C. glabrata* and *C. krusei* has been seen in the post FCZ era and in settings with azole selection pressure [31]. Worldwide, there is a variation in the distribution of *Candida* spp. identified from vaginal swabs and depends largely on the location as well as the population studied (Fig. 1).

Treatment of vaginal candidiasis is successfully achieved by use of azoles [32]. NCAC related disease is less likely to respond to azole therapy, alternative treatment with AMB suppositories with or without topical azole is recommended. In the current study, isolates showed high susceptibility to AMB (97.5%) and this observation has been corroborated by studies done in various other countries including Lebanon [30, 33–35]. Resistance rates of *C. albicans* to VCZ, FCZ, and ICZ and in this study were 2.5, 10, and 12.5%, respectively, which are in contrast to earlier data from Lebanon reporting 3 to 6%, 0 to 6, and 38% resistance, respectively [30]. However, despite high susceptibility rates against FCZ and VCZ in the previous study, their MIC_90_ showed an elevated trend over 10 year of study period [30]. The increase in azole resistance in our study can be attributed to the frequent empiric prescription of FCZ for sporadic VVC, which may result in FCZ-resistant *C. albicans* causing recurrent VVC infection to emerge [36]. Identification of the most common molecular mechanism of resistance among our clinical isolates would help in understanding if there is any spread of resistance gene between *C. albicans* and NCAC. Since through vertical or horizontal transmission, 5–30% of all colonized preterm neonates may develop invasive *Candida* infection [13–15], prophylaxis with antifungal agents in this group of patients has proven effective in preventing such an infection. However, an increase in MIC against antifungal agents may have major consequences resulting in poor outcomes and higher mortality rate among neonates with invasive *candida* infection.

Although treatment of asymptomatic pregnant women with *Candida* colonization in the genital tract is not yet recommended, some countries such as Germany have started to implement the process of screening and treatment of women found to be colonized vaginally by *Candida* spp. or those who present with VVC in the third trimester [37]. In Lebanon, unlike group B streptococcus (GBS), routine screening for the presence of *Candida* spp. in pregnant women in the third week of gestation is not considered as part of a routine surveillance by the obstetricians. Since invasive candidiasis in neonates is becoming a serious and common cause of late onset sepsis, with mortality rates reaching as high as 25–35% [10], screening simultaneously for both GBS and *Candida* spp. in pregnant women would reduce the rate of sepsis, meningitis, oral thrush and diaper dermatitis in newborns with these organisms acquired during vaginal delivery.

It is reported that vaginitis in pregnancy is related to adverse perinatal outcome [38]. In the current study, we aimed to find correlation between the presence of candidiasis and pregnancy outcome. Our results showed that height decreased with the presence of *Candida* species. This reduction was statistically significant in the presence of *C. krusei or C. glabrata.* However no effect was observed on the weight of the baby. This finding was consistent with a study done previously in Iran where they found no association between vaginal *Candida* colonization and low birth weight [39]. The current study has also shown that *Candida species* cause gestational complications which is also in agreement with a previous study done in China [40].

Among the different studied variables which may be affected by *Candida,* such as gestational complications, gestational diabetes, vaginal discharge, induced labor and recurrent UTI, the present study confirmed that the presence of *C. albicans* was significantly associated with women with gestational diabetes and both *C. krusei and C. glabrata* on women with gestational complications. Future case control studies should be performed to compare the clinical outcome of pregnant women infected with any microorganism versus non infected women.

The study has potential limitations which include the lack of screening of other pathogens in pregnant women; these may have an impact on pregnancy outcomes. Case control studies should be also implemented to determine if exposure to C*andida* species has an association with pregnancy outcomes.

## Conclusions

In conclusion, increasing rates of NCAC strains among pregnant women in Lebanon should be looked at as both novel and alarming. Extensive surveillance studies should be done on all clinical specimens yielding significant growth of *Candida* spp. and the effect of resistance pattern on ICI. As a consequence of selective pressure, emergence of drug resistance is inevitable. Therefore future studies should focus on the emergence of drug-resistant *Candida* strains and their frequencies. The susceptibility pattern of *C. albicans* to antifungal agents varies with region and would require constant monitoring of any unusual increase in resistance.

## Data Availability

The datasets used and/or analysed during the current study are available from the corresponding author on reasonable request.

## Abbreviations

AMB: Amphotericin B
Beta: Standardized beta coefficients
CI: Confidence interval
CLSI: Clinical and Laboratory Standards Institute
FCZ: Fluconazole
GBS: Group B streptococcus
ICZ: Itraconazole
NCAC: Non-*C. albicans Candida*
NICU: Neonatal intensive care unit
R2: Coefficient of determination
SDA: Sabouraud dextrose agar
UTI: Urinary tract infections
VCZ: Voriconazole
VVC: Vulvovaginal candidiasis
